# Investigating Polypharmacology through Targeting Known Human Neutrophil Elastase Inhibitors to Proteinase 3

**DOI:** 10.1021/acs.jcim.3c01949

**Published:** 2024-01-26

**Authors:** Parveen Gartan, Fahimeh Khorsand, Pushpak Mizar, Juha Ilmari Vahokovski, Luis F. Cervantes, Bengt Erik Haug, Ruth Brenk, Charles L. Brooks, Nathalie Reuter

**Affiliations:** † Department of Chemistry, University of Bergen, Bergen 5020, Norway; ‡ Computational Biology Unit, University of Bergen, Bergen 5020, Norway; § Department of Biomedicine, University of Bergen, Bergen 5020, Norway; ∥ Core Facility for Biophysics, Structural Biology, and Screening, Department of Biomedicine, University of Bergen, Bergen 5020, Norway; ⊥ Department of Medicinal Chemistry, College of Pharmacy, University of Michigan, Ann Arbor, Michigan 48109, United States; # Centre for Pharmacy, University of Bergen, Bergen 5020, Norway; 7 Department of Chemistry, University of Michigan, Ann Arbor, Michigan 48109, United States; 8 Biophysics Program, University of Michigan, Ann Arbor, Michigan 48109, United States

## Abstract

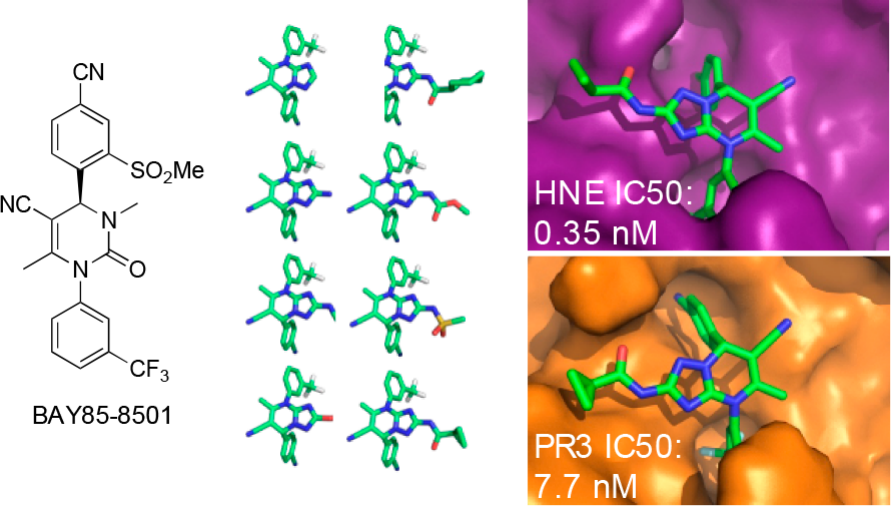

Using a combination of multisite λ−dynamics (MSλD) together with *in vitro* IC_50_ assays, we evaluated the polypharmacological potential of a scaffold currently in clinical trials for inhibition of human neutrophil elastase (HNE), targeting cardiopulmonary disease, for efficacious inhibition of Proteinase 3 (PR3), a related neutrophil serine proteinase. The affinities we observe suggest that the dihydropyrimidinone scaffold can serve as a suitable starting point for the establishment of polypharmacologically targeting both enzymes and enhancing the potential for treatments addressing diseases like chronic obstructive pulmonary disease.

Using a combination of computational prediction and in vitro IC_50_ assays, we demonstrate the polypharmacological potential of a scaffold that has already led to inhibitors of the human neutrophil elastase, some of which are currently in clinical trials targeting cardiopulmonary disease.

Neutrophils are the first line of defense against infection by invading pathogens. They are also important players in the regulation of inflammation.^1−3^ In the last 30 years, both academic and industrial actors have devoted large efforts in developing drug-like compounds inhibiting the activity of the human neutrophil elastase (HNE),^4,5^ a neutrophil serine protease (NSP).^4^ Imbalance between HNE and its endogenous inhibitors results in HNE overactivity and has been linked to several cardiopulmonary diseases including chronic obstructive pulmonary disease,^4^ as well as other chronic inflammatory conditions. Several promising compounds have recently progressed to clinical trials. To date, Sivelestat (ONO-5046, Elaspol) is the only nonpeptidic HNE inhibitor having reached the market but with mitigated results.^6^

Proteinase 3 (PR3) is another NSP whose pathophysiological role is close to that of HNE. PR3 has also been identified as a drug target (though more recently than HNE), suggesting that dual inhibition of both enzymes should be beneficial for a number of pathologies.^7^ While a few nonpeptidic inhibitors of PR3 have been reported, none have progressed to clinical trials. The resemblance of the two enzymes in terms of sequence (57% sequence identity) and structure suggests that existing HNE inhibitors could form the basis for dual inhibitors of both HNE and PR3 despite them having somewhat different substrate specificity.^8^ This motivated our investigations into whether inhibitors identified to be active against HNE could serve as polypharmacological agents to PR3. We investigated 11 of the latest noncovalent elastase inhibitors based on the pyridone and dihydropyrimidinone lead structures from Bayer HealthCare AG (listed in Table 1).^9−11^ The dihydropyrimidinone scaffold was designed and explored in different directions (e.g., with the triazolopyrimidine scaffold^10^) to overcome the limitations of previous generations of compounds^12^ (Figure 1, Table 1). Ultimately, this scaffold resulted in several selective HNE inhibitors that progressed to clinical trials. Examples include BAY85-8501^9^ (Compound **1** in Table 1) and CHF6333^13,14^ from Bayer Healthcare and Chiesi Farmaceutici, respectively. Compounds **2**–**11** listed in Table 1 were selected because they contain a triazolopyrimidine (and its substituent R) extending the compounds toward the S2, S3, and S4 subsites of HNE and PR3. These subsites are known to be different between HNE and PR3 notably with the L99K substitution in PR3.

**Table 1:**
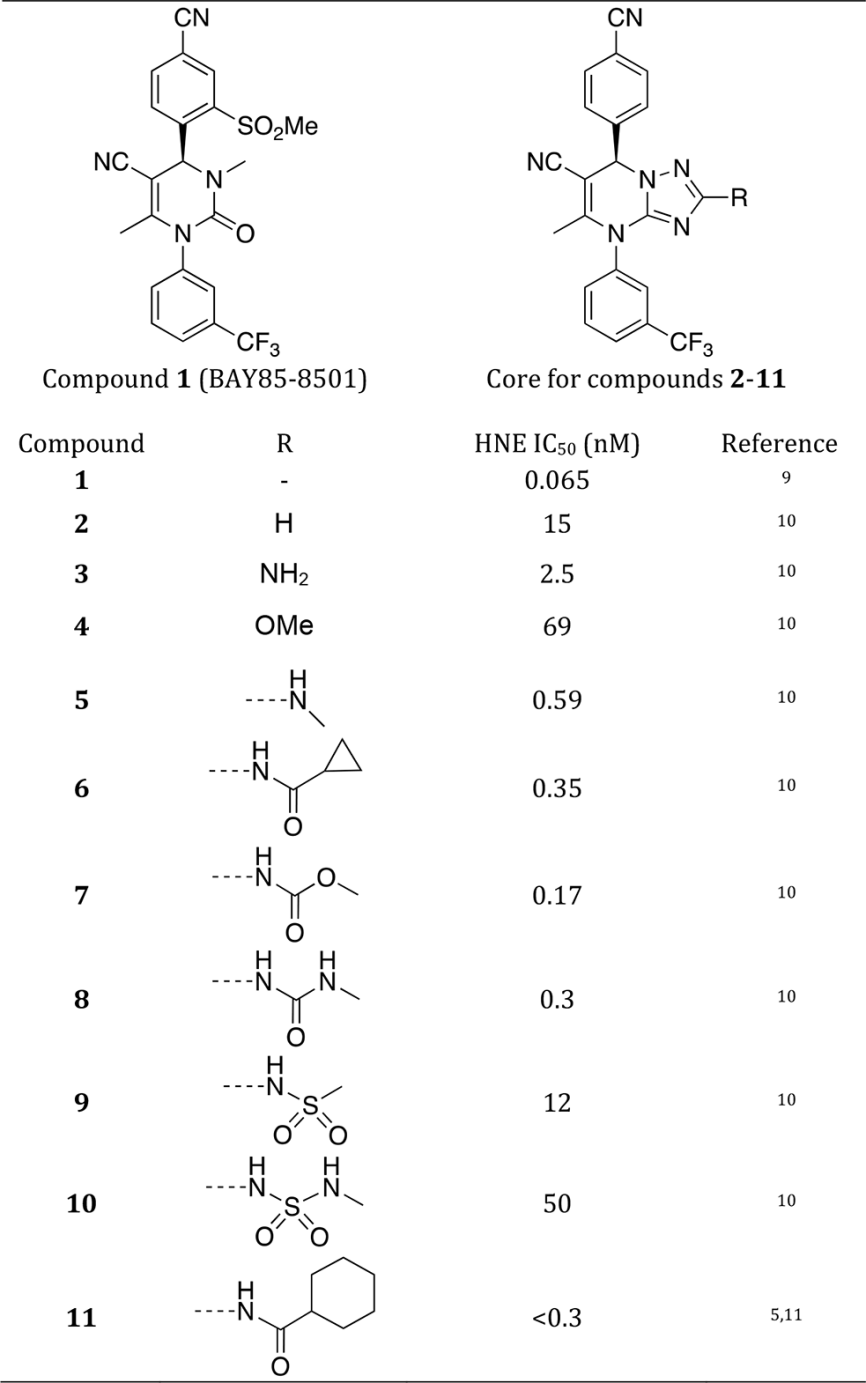
Bayer HealthCare AG Compounds:^9−11^ Structure of the 11 Compounds Used and of the Core Used for MSλD Calculations on Compounds **2-11**

**Figure 1:**
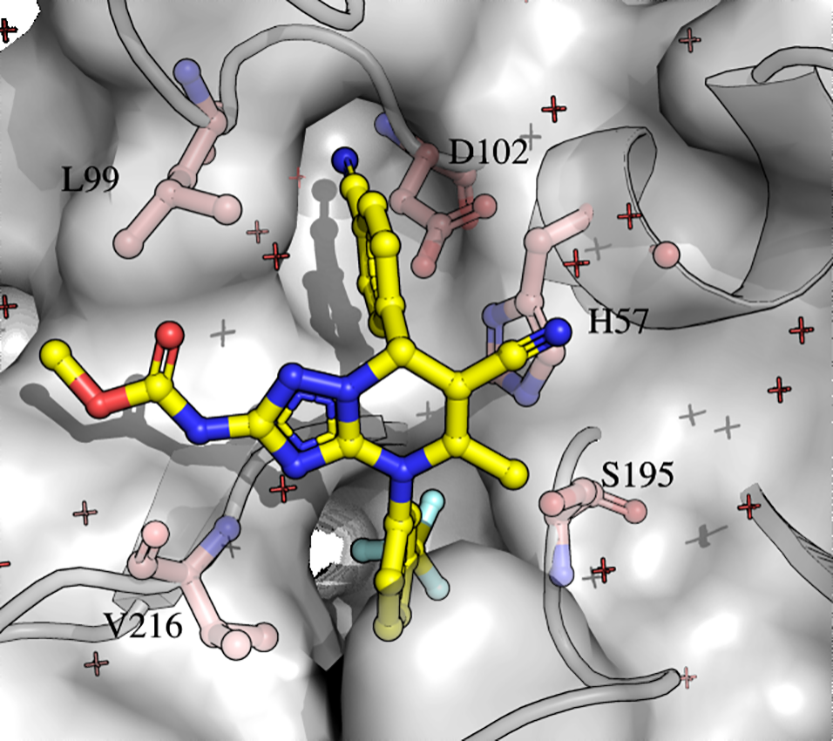
Triazolopyrimidine extension of the dihydropyrimidinone scaffold (compound **7** in Table 1) in the active site of HNE (X-ray structure, PDB ID:5a8y; data from ref (10)). The backbone and surface of the HNE ligand binding sites are represented in gray, and the side chains of amino acids in the catalytic (H57, D102, S195) and ligand-binding sites (V216, L99) are represented with light pink sticks. The dihydropyrimidinone is shown in sticks and colored by atom types (C, yellow; N, blue; O, red; F, light blue). The figure was generated by using PyMol.^15^

To short-circuit long and tedious syntheses and the establishment of assays, we decided to utilize computational free energy methods to predict the binding affinities for PR3 and inform the *in vitro* testing program. It has been well demonstrated through numerous studies that computational free energy methods utilizing the current generation of molecular mechanics force fields show good correlations with measured binding affinities.^16−18^ One approach, used here, the multisite λ-dynamics methodology,^19−21^ provides much higher throughput^22^ in its demonstrated ability to simultaneously explore combinatorial chemical spaces comprised of multiple sites of derivatization on a template.^22^

We thus define a multiple topology model (MTM) consisting of a single perturbation site for compounds **2**–**11** with a common core as shown in Table 1 and another dual-topology system for compounds **1** and **2** with NOE based tethering (Cf. the Supporting Information). Multisite λ-dynamics (MSλD) in the CHARMM^23,24^ package was utilized to predict binding affinities of the HNE inhibitors shown in Table 1.

To ensure we had the best models to represent the selected ligands and their interactions with the protein, we explored four different force field combinations for protein and ligands (Table 2) and compared the predicted values to published experimental data for HNE. Details regarding the compound parameter sets, MSλD system setup, and molecular dynamics simulations can be found in the Supporting Information. The predicted free energies obtained with MSλD are provided in Table S3 together with the free energies derived from published experimental data. The experimental binding affinities were calculated from the IC_50_ values listed in Table 1 using Δ*G* = RTln(IC_50_) at 298.15 K. Since the measurements were carried out at substrate concentrations significantly below K_m_, the binding constant K_i_ can be approximated by the IC_50_ value. Based on repeated trials of the free energy calculations, the MSλD calculations are well converged, as indicated by the low uncertainties that we observed. Our overall comparison between the MSλD predicted binding free energies and the experimental data is shown in Figure 2. The Pearson’s correlation coefficient (R) between experimental values and predicted binding free energies arising from different combinations of force fields varies between 0.4 and 0.9. The poorest correlation among the four selected force fields is from the use of the CHARMM/CGenFF combination (R = 0.4). This combination also shows the highest root-mean-square error (RMSE: 1.6 kcal/mol) between the predicted and measured binding affinities. It is followed by AMBER/GAFF2 with a correlation of R = 0.8 and an RMSE of 0.9 kcal/mol. Both CHARMM/CGenFF and AMBER/GAFF2 have three common outliers, compounds **9**, **10**, and **11**. Compounds **9** and **10** both contain a sulfonyl group, whereas compound **11** has a cyclohexyl substituent. CGenFF underestimates the binding affinity for compound **6** which contains a three-membered cyclopropyl ring. Using OPLS/OPLS, all four outliers are within 1 kcal/mol of their corresponding experimental data, and the correlation over all compounds is good (R = 0.8) with an RMSE of 0.8 kcal/mol. AMBER-GAFF2 and OPLS–OPLS yield RMSE below 1 kcal/mol, but the predictions with CHARMM/OPLS yielded the best correlation (R = 0.9) and the lowest RMSE (0.4 kcal/mol) with all predicted binding affinities falling within 1 kcal/mol of the experimental data.

**Table 2:** Force Fields Used to Represent the Protein and Compounds^a^

Short name	Protein	Compounds parameter set
CHARMM-CGenFF	CHARMM36m^27^	CGenFF
Amber-GAFF2	Amber ff14SB^28^	AM1-BCC and GAFF2
OPLS–OPLS	OPLS-AA^29,30^	1.14*CM1A-LBCC and OPLS-AA
CHARMM-OPLS	CHARMM36m	1.14*CM1A-LBCC and OPLS-AA

a All systems were solvated with the TIP3P^25,26^ water model and 0.15 M KCl (except for OPLS–OPLS where we used NaCl).

**Figure 2:**
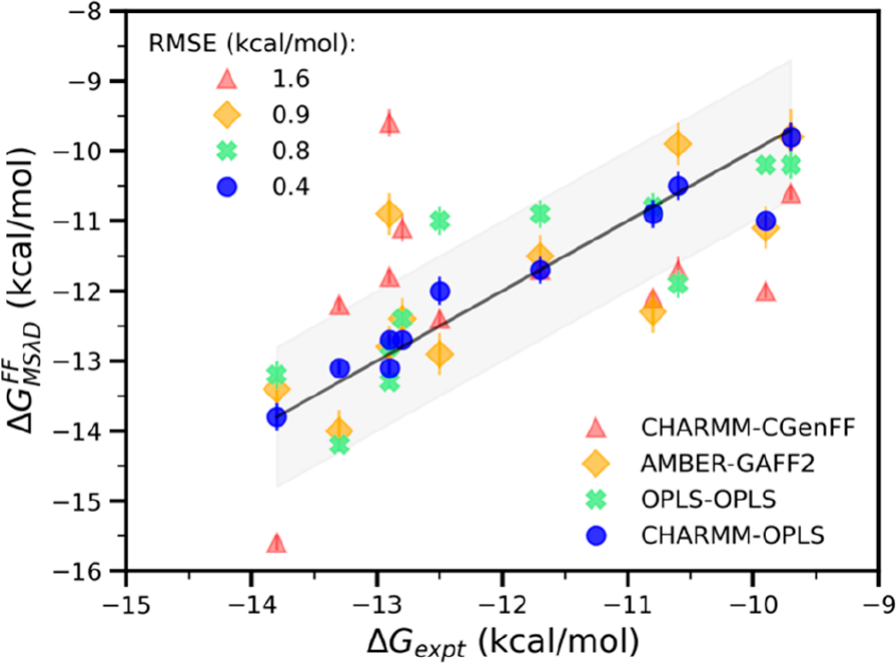
Experimental vs MSλD predicted binding free energies for HNE with different combinations of force fields. The experimental values were taken from von Nussbaum et al.^9−11^ Correlation of MSλD predicted free energies with experimental data varies with the force field combination. Pearson’s correlation for the different combinations of force fields varies from R = 0.4 (CHARMM-CGenFF) to 0.8 (OPLS–OPLS, AMBER-GAFF2) and up to R = 0.9 (CHARMM-OPLS).

As we aim at predicting the affinity of the 11 compounds for PR3, we then calculated their relative binding affinities for PR3. Following the benchmarking results obtained for HNE, we chose the CHARMM/OPLS force field combination. The predicted relative binding free energies (ΔΔ*G*_msλd_^*FF*^) obtained for PR3 from MSλD using eqs 1–2 (Cf. the Supporting Information) are reported in Table S4. The MSλD values are also very well converged and again have low uncertainties. To compare the predicted potency of each compound between the two enzymes, we needed to calculate their absolute binding free energies (Δ*G*_*msλd*_^*FF*^). To that goal, we purchased compound **1** (BAY85-8501) and determined its IC_50_ for HNE and PR3. We used the assay reported in Budnjo et al.^31^ with minor modifications (Cf. the Supporting Information). For PR3, the reaction was initiated by adding the FRET peptide (Abz-VADnVADYQ-EDDnp, excitation filter 320, emission filter 420) while the HNE reaction was started by adding the fluorogenic substrate (MeOSuc-AAPV-AMC, excitation filter 360, emission filter 460), both at a final concentration of 5 μM. Our IC_50_ value for HNE (0.5 nM) is somewhat higher than reported by Nussbaum et al.^9^ but still within the nanomolar range. The measured IC_50_ for PR3 is 200-fold higher (101 nM) (Table 3). We used these values as anchors to convert the MSλD relative free energies for all compounds to absolute free energies for both HNE and PR3 (eq 3 in the Supporting Information). The resulting values (Tables S5–S6) predict that all the selected Bayer compounds have relatively lower binding affinities for PR3 as compared to HNE. The predicted potencies for PR3 vary in a range characteristic of lead compounds (IC_50_ between 37 nM and 23 μM).

**Table 3:** Experimentally Determined IC_50_ Values for Both HNE and PR3^a^

	IC_50_ (nM)		pIC_50_		IC_50_ ratio
Compound	HNE	PR3	HNE	PR3	(PR3/HNE)
**1** (BAY 85-8501)	0.5 ± 0.1	101.0 ± 9.0	9.3	7.0	202
**5**	1.3 ± 0.2	190.0 ± 10.0	8.9	6.7	146
**6**	0.6 ± 0.0	7.7 ± 0.6	9.1	8.1	13
**7**	1.1 ± 0.1	21.3 ± 2.1	8.9	7.7	19
**8**	0.8 ± 0.1	58.9 ± 6.0	9.1	7.2	74

a The selectivity of the compounds is reported as the ratio of PR3 IC_50_ values with respect to the HNE IC_50s_.

We then went on to experimentally determine the IC_50_s for compounds **5**-**8** against both enzymes as described above for Compound **1**. Compounds **5**-**8** were selected because of their high predicted potency against PR3. The measured IC_50_s for both HNE and PR3 corresponding to the best enantiomer in each case are reported in Table 3. We note that our IC_50_s for HNE are all slightly higher than those reported in the literature but are nevertheless in good agreement. The measured IC_50_s of these compounds toward PR3 are higher than HNE, as predicted but still in the nanomolar range (7 nM to 190 nM). The selectivity for PR3 relative to HNE is lower than the third and fourth generation compounds that had IC_50_ (or *K*_i_) ratios of more than 1900 and 600, respectively.^12,32^

We then took advantage of our experimental IC_50_s to evaluate the accuracy of the four different combinations of force fields on PR3 affinity prediction, as was done for HNE (Figure 2). The MSλD predicted binding affinities for PR3 are compared to the experimental values in Figure 3. The RMSE between experimental and predicted values from the four different combinations is ca. 1 kcal/mol. Both CHARMM/CGenFF and CHARMM/OPLS yield RMSEs equal to 0.8 kcal/mol. With CHARMM/OPLS, the RMSE is higher than the 0.4 kcal/mol obtained for predictions of affinity of the compounds for HNE. This may be attributed to the lack of a small molecule bound X-ray structure for PR3 and the need to instead use a computational model of the complex as the starting structure of the MSλD calculations. Moreover, the range of experimental binding affinities and the number of data points might also partly explain the statistics obtained. Overall, our work shows that MSλD ΔΔ*G* predictions with the right combination of force fields and a good X-ray structure as starting conformation give values within 1 kcal/mol from experimental values. Our observation that the CHARMM-OPLS combination yields results in good agreement with experimental data is consistent with the work of Vilseck et al.^22^ who showed for beta secretase 1 (BACE1) that using CM1A charges in combination with CGenFF and CHARMM provides superior agreement with experimental data. Further validation on large data sets is required to evaluate the performance of force field combinations.

**Figure 3:**
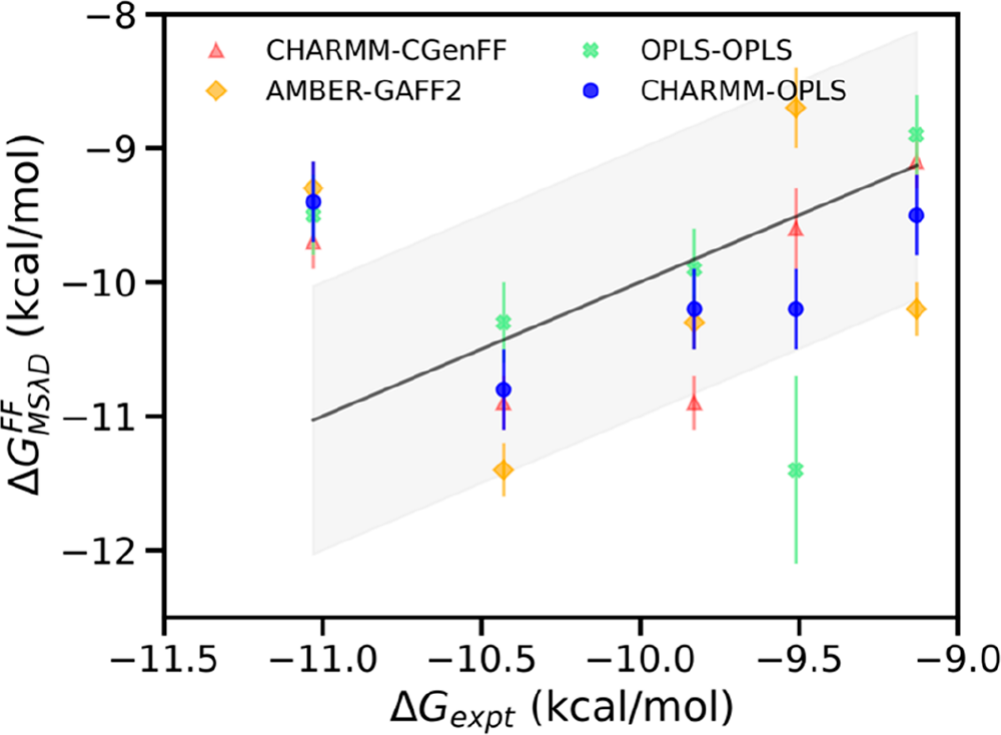
Experimental vs MSλD predicted binding free energies for PR3 (Compounds **1** and **5**–**8**). The Δ*G*_*MSLD*_^*FF*^ and Δ*G*_*expt*_ values are available in Table S8.

Finally, we compared the predicted potency of the compounds for the two enzymes to evaluate the potential of the scaffold to produce dual inhibitors of PR3 and HNE (Figure 4). Overall, the 11 compounds are predicted to have a potency for PR3 lower than that for HNE, in agreement with our experimental data for five of these compounds (Table 3). Their predicted selectivity is also in line with their development as specific HNE inhibitors.^9,10^ Given that some compounds show a fairly low IC_50_ ratio *in vitro* (Table 3), we propose that the dihydropyrimidone and triazolopyrimidine scaffolds can be used to produce highly potent PR3 inhibitors. This entails a more in-depth exploration of the structure activity relationship using, for example, a larger number of compounds and exploiting the differences in subsites between HNE and PR3.^33,8,31^ Our results thus provide data relevant for optimization around the dihydropyrimidone and triazolopyrimidine scaffolds to produce either PR3 specific inhibitors or dual inhibitors with low IC_50_ ratios. The latter have the potential to increase the efficacy of drug candidates targeting selectively HNE.

**Figure 4:**
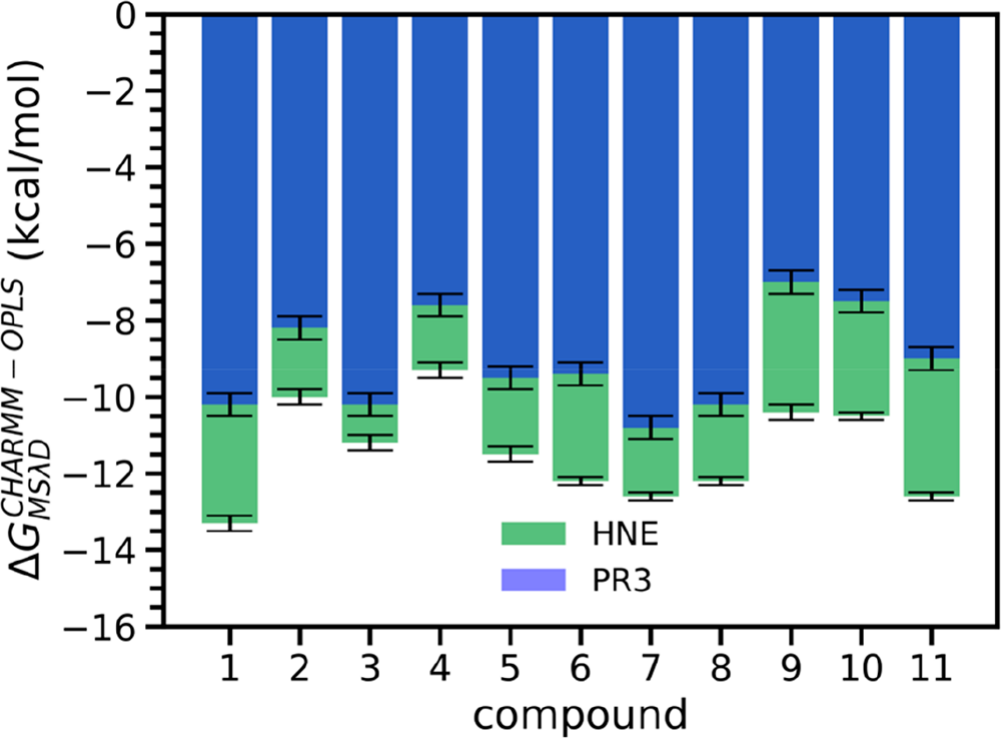
Comparison of MSλD predicted binding free energies between HNE and PR3. The relative binding free energies from MSλD (Δ*G*_*MSLD*_^*CHARMM* – *OPLS*^) are available in Tables S9 and S10.

## Data Availability

All data and software used in this study are available freely. Data sources and identifiers are given in the text. Moreover simulation files are available at https://github.com/reuter-group/bayer_compounds_msld). The repository contains ligands mol2 files along with original FF parameters from CGenFF, GAFF2, and OPLS; MSλD prep containing hybrid ligands, ALF biases, and CHARMM production run files used for relative binding free energy calculations; CHARMM input files for bookending corrections.

## Supporting Information Available

Computational and experimental details including IC_50_ assays and compound syntheses. Detailed results and tables reporting predicted relative and absolute binding free energy for HNE and PR3 with the four combinations of force fields (PDF)
Additional file (spreadsheet) is provided containing the experimental IC_50_ data and the corresponding free energies of binding, as well as MSLD computed relative binding free energies with renormalized charges (CRN) and the MBAR free energies associated with perturbation of original force field charges (FF) to renormalized charges (CRN) (XLSX)
