# Supplementary material for: Investigating Polypharmacology through Targeting Known Human Neutrophil Elastase Inhibitors to Proteinase 3
Source: J Chem Inf Model. 2024 Jan 26;64(3):621–6. doi: 10.1021/acs.jcim.3c01949 (PMC10865350; doi:10.1021/acs.jcim.3c01949)
Supplement: Supplementary file 1 — ci3c01949_si_001.pdf [file ci3c01949_si_001.pdf]

Supporting information

Investigating Polypharmacology Through Targeting Known Human Neutrophil Elastase Inhibitors to Proteinase 3

Parveen Gartan[1,2], Fahimeh Khorsand[3], Pushpak Mizar[1], Juha Ilmari Vahokovski[4], Luis F. Cervantes[5], Bengt Erik Haug[1,6], Ruth Brenk[3], Charles L. Brooks III[7,8*], Nathalie Reuter[1,2*]

[1]Department of Chemistry, University of Bergen, Bergen, 5020, Norway
[2]Computational Biology Unit, University of Bergen, Bergen, 5020, Norway
[3]Department of Biomedicine, University of Bergen, Bergen, 5020, Norway
[4]Core facility for Biophysics, Structural Biology, and Screening, Department of Biomedicine, University of Bergen, Bergen, 5020, Norway
[5]Department of Medicinal Chemistry, College of Pharmacy, University of Michigan, Ann Arbor, Michigan, 48109, USA
[6]Centre for Pharmacy, University of Bergen, Bergen, 5020, Norway
[7]Department of Chemistry, University of Michigan, Ann Arbor, Michigan, 48109, USA
[8]Biophysics Program, University of Michigan, Ann Arbor, Michigan, 48109, USA

* Corresponding authors: brookscl@umich.edu, nathalie.reuter@uib.no

I. Modeling and computations

I.1. Computational details

System setup. Structure for Human Neutrophil Elastase (HNE) in holo form was obtained from PDB ID 5A8X (method: Xray, resolution 2.23 Å). The glyocsylations were removed from 5A8X using PyMol.[1] The initial structure of HNE lacks three residues (ARG146, ASN147, ARG148) which were modelled using Modeller[2] 9.22 web service in Chimera.[3] The resulting final structure has 218 residues. The protonation states of histidine were chosen based on visual inspection of the surroundings (25, 40 and 57 were protonated on δ-N whereas 71 and 210 were protonated on ϵ-N).[4] Other titratable groups were in their standard protonation state following pKa prediction with PropKa.[5]

A monomer of PR3 in apo form was extracted from the tetrameric structure in PDB 1FUJ (method Xray, resolution 2.20 Å). Since the chains A to D in the tetramer are extremely similar (RMSD 0.4 Å) the results of MD simulations should not be affected by the choice of chain. Hence, we arbitrarily chose chain A. The histidine residues numbered 24, 48, 132 were protonated on their ε-N and 20, 40, 57, 71, 82, 147, 179 were protonated on δ-N. The aspartic acid 213 was protonated at OD1 based on earlier pKa calculations[6] and in agreement with Fujinaga et al.[7]

Docking. The protein pdbqt files were prepared using AutoDock Tools. Only polar hydrogens were added to the protein. The grid box center was placed on the center of protein and a size of 40*40*40 (spacing 1 Å) was used. All of the ligand structures (**Table 1**) were drawn in Marvin Sketch (v. 20.9)[8] with explicit hydrogen atoms, using the stereochemistry shown in **Table 1,** and saved as mol2 files. Open Babel 2.4.1[9] was used to prepare ligand pdbqt files. AutoDock Vina[10] was used to dock the ligands in protein pocket using blind docking for both HNE and PR3 with default exhaustiveness of 8.

Small molecule parameters. The mol2 files from Marvin Sketch were used for parameter assignments using the force fields and programs detailed in **Table S1**. Open Babel and PyMOL were used to interconvert between mol2 and pdb formats.

Table S1. The three different parameter sets used to represent small molecules for relative binding free energy calculations.

Parameter set	Assignment program	Force Field
CGenFF	CGenFF v 2.3.0,[11,12] Silcsbio package (v. 2020.1.4)	CGenFF v4.3[13,14]
AM1-BCC/GAFF2	AmberTools22,[15] Antechamber[16]	GAFF2 v2.11[17]
1.14*CM1A-LBCC/OPLS-AA	LigParGen[18–20] webserver (accessed: 23 May 2023) or standalone (with BOSS[21] v 5.0)	OPLS-AA[22,23]

AM1-BCC = Austin Model 1-bond charge correction

LBCC = localized bond-charge correction

Multisite λ dynamics (MS*λ*D). The set of ligands is distributed into one reference or core and different sites containing different substituents. First, compounds **2-11** are aligned using Chimera[24] and then, using the Maximal Common Substructure (MCS) algorithm with a RMSD cut-off of 0.8 Å for core atoms, the reference (core; **Table 1**) and different substituents (R group; **Table 1**) are generated. The resultant hybrid molecule contains a single copy of the core atoms and different substituents at different sites. Next, charge renormalization (CRN) is performed so that the different substituents at a single site have the same charge and the net charge of core atoms + each substituent at different sites should be zero (all compounds were neutral). The same procedure is used here for charge renormalizations as described by Vilseck et al.[25] This step uses the charges assigned from the programs listed in **Table S1**. In the final step, CHARMM[26,27] rtf and prm files are generated for the hybrid molecule where the parameters (CGenFF/OPLS/GAFF2 atom types, angles, dihedrals, bond connectivity) are copied from the rtf and prm file of individual molecules. Another dual topology[28,29] system was prepared for compounds **1** and **2** to use compound **1** as an anchor due to its commercial availability.

The hybrid ligand for each different combination of force fields (**Table 2**) is solvated in a cubic water box of TIP3P water molecules (cutoff 12 Å) using the MMTSB toolset.[30] A protein-hybrid ligand system is also prepared using the four different combinations of force fields (**Table 2)** and preserving the crystal water molecules in the respective crystal structures of HNE and PR3. These systems were also solvated using CHARMM 45a2 with preequilibrated TIP3P water molecules in a cubic box extending 10 Å from the longest axis of the protein-ligand complex. To neutralize the protein-hybrid ligand system, K^+ (or Na^+) and Cl^- ions corresponding to a concentration of 0.15 M KCl or NaCl (see **Table 2** in main text). NaCl was used for simulations with the OPLS-OPLS combination as K^+ ion parameters are not available in the CHARMM compatible OPLS-AA protein parameter files. Ions were added by randomly replacing bulk water molecules. To run the multisite λ dynamics simulations the systems were prepared using the BLOCK module in

CHARMM program. The different angles and dihedrals were removed between alchemical groups at a particular site. The core atoms and different substituents were each assigned a block and then their own coupling parameter i.e., λ. The input parameters for each λ such as initial λ value, velocity, mass, and reference free energy (or biasing potential) were assigned using LDIN. The bonds, angles, and improper dihedrals were excluded from scaling with λ. MSλD was initialized along with defining the total number of blocks and the function form of λ (FNEX = 5.5[31]). In this functional form, λ is made a function of θ and it is θ that has fictitious masses and is propagated through the dynamics. Finally, the total number of biasing potentials were defined using LDBI for all the different substituents excluding the core. Each λ/substituent has four different biases (fixed, skew, quadratic, and end) applied to it at any point. The functional form and details about the biases can be found elsewhere.[32,33] CHARMM NOE based tethering was used for compound **1** and **2** to ensure that the non-interacting ligands stay in the binding site. The pairs of atoms between compounds **1** and **2** for assigning the NOE restraints were picked by visual inspection. Eight atoms (four each) were picked from compound **1** and **2**.

The simulations were performed with CHARMM 45a2 using the domain decomposition (DOMDEC) method or CHARMM 47a2 using BLaDE[34] on graphical processing units (GPUs). The systems were minimized using 200-450 steps using the steepest descent algorithm. Molecular dynamics simulations were performed in the isothermal–isobaric ensemble (NPT) at 298.15 K and 1 atm using leap integrator, Nose-Hoover thermostat for temperature control and Langevin piston[35] (friction coefficient: 10 ps^{-1}) for pressure control; BLaDE utilized a Langevin thermostat for temperature control and a Monte Carlo barostat for pressure control (move attempts: 100 steps) with a friction coefficient of 0.1 ps^{-1}. An integration time step of 2 fs was used, trajectory frames were saved every 2 ps and λ values saved every 20 fs. Heavy atom-hydrogen bond lengths were constrained with SHAKE.[36] Periodic boundary conditions (PBC) along with particle mesh Ewald (PME) for long range electrostatics were used. A non-bonded cutoff of 12.0 Å along with truncation (using VFSwitch) of van der Waals interactions between 9-10 Å was employed. To avoid the end point singularities, soft-core potentials were used.[32]

One final adjustment in the underlying force fields pertains to the use of the scaling of electrostatic interactions between atoms interacting through three bonds, i.e., the atoms in dihedrals (e14fac in CHARMM). The force fields suggest different values (AMBER/GAFF2: 0.8, OPLS: 0.5, CHARMM/CGENFF: 1.0) but we used combinations of force fields. Since CHARMM only supports one universal e14fac, and because the CHARMM/BLaDE interface does not currently support e14fac different than 1, we adjusted this value as indicated in the table below, using e14fac = 1 for all simulations. We tested that this had little effect on the conformational sampling

of proteins and ligands, as the e14fac is expected to affect sampling of the torsion space. We find that the protein RMS fluctuations are similar regardless of whether e14fac=1 or e14fac=(0.5,0.83) are used in OPLS and Amber, the same applies to the ligands in water. The protein RMSD relative to the starting structures showed slight differences, but all RMSD averages were below 2.0 Å. Thus, we conclude that this small change is unlikely to significantly affect our free energy calculations.

1-4 interaction scaling (CHARMM keyword - e14fac).

Force Field	Default values	MSλD simulation		Method
		HNE	PR3	
CHARMM-CGenFF	1.0 (protein and ligands	1.0	1.0	domdec
AMBER-GAFF2	0.8 (protein and ligands)	1.0	1.0	BLaDE
OPLS-OPLS	0.5 (protein and ligands)	1.0	1.0	BLaDE
CHARMM-OPLS	1.0 (protein) 0.5 (ligands)	1.0	1.0	BLaDE

MSλD simulations are divided into four phases. The first three phases are used for the optimization of biases applied on each substituent and to monitor sampling of each substituent, where the objective is to maximize the transitions between substituents. Only after the λ landscape has flattened are production simulations run. The first phase consists of running several short ~100 ps simulations to obtain a rough estimate of the biases. In the second phase several longer ~1 ns simulations are used to refine the biases followed by phase three of 5 independent simulations in 5 duplicates for sufficient sampling of each substituent. After every simulation in the initial two phases, all of the biasing potentials for each substituent were estimated from the relative free energy differences, which were calculated using the weighted histogram analysis method[37] (WHAM). This iterative method was used to obtain new biases for subsequent steps. To avoid systematic biases in the simulations, the restart runs were chosen randomly for the first two phases. Production simulations were run for 30 ns in five duplicates with a cumulative sampling time of 150 ns. The first 5ns was considered equilibration and the analysis was made on the remaining 25 ns. For the ligand in solvent system, the production run accounted for 100 ns (20 ns x 5), with 1 ns as the equilibration in each duplicate. The biases were

subtracted when calculating the final relative free energy differences. The NOEs used between compounds **1** and **2** had no direct effect on the populations.

The final relative binding free between two different ligands/substituents L2 and L1 can be calculated as[31,38]

$$\Delta\Delta G^{CRN}_{ms\lambda d}(L_1^{\#} \rightarrow L_2^{\#}) = -k_B T\, ln \frac{P\,(\lambda_{L_2^{\#}}=1)}{P\left(\lambda_{L_1^{\#}}=1\right)} \tag{1}$$

# - represents that the ligands carry renormalized charges.

MSλD bookending charge corrections.[25] The MSλD relative binding free energy calculations between compounds **2-11** were performed with renormalized charges. To obtain the binding free energies corresponding to the original force field (FF) charges we performed single step charge perturbations (SSP) using fixed λ(=1). We ran MD simulations for ligands in water and protein-ligand complex states with renormalized charges (CRN) and the original FF charges using a similar molecular dynamics (MD) simulations protocol as was used for MSλD simulations. These calculations were run for 10 ns each in addition to the MSλD production simulations. The MBAR module in the pymbar.py[39,40] python package was used to calculate the relative binding free energies. The relative binding free energy corresponding to the original FF charges is calculated as:

$$L_1 \xrightarrow{\Delta G_{SSP}^{FF\rightarrow CRN}(L_1)} L_1^{\#} \xrightarrow{\Delta\Delta G^{CRN}_{ms\lambda d}\,(L_1^{\#}\rightarrow L_2^{\#})} L_2^{\#} \xrightarrow{\Delta G_{SSP}^{FF\rightarrow CRN}\,(L_2)} L_2$$

$$\Delta\Delta G^{FF}_{ms\lambda d}(L_1 \rightarrow L_2) = \Delta\Delta G^{CRN}_{ms\lambda d}\left(L_1^{\#} \rightarrow L_2^{\#}\right) + \Delta G_{\mathrm{SSP}}^{FF\rightarrow CRN}\,(L_1) - \Delta G_{\mathrm{SSP}}^{FF\rightarrow CRN}\,(L_2) \tag{2}$$

Where, $L_{1/2}$ – ligands represented with original FF charges

$L^{\#}_{1/2}$ - ligands represented with renormalized charges

SSP – single step perturbation

Charge corrected absolute free energies. The absolute binding free energies ($\Delta G^{FF}_{ms\lambda d}$) are calculated from the MSλD relative binding free energies ($\Delta\Delta G^{FF}_{ms\lambda d}$) and the experimental binding affinities (ΔG_{expt}).[41] To minimize systematic error between computation and experiment when choosing one reference compound, we sought to find a constant **A** such that we minimize the variance between the relative binding affinities.

$$\sigma^2_{min} = \sum_{i=1}^{n}\left[\Delta\Delta G^{r-i}_{expt} - \left(\Delta\Delta G^{r-i}_{ms\lambda d} + A\right)\right]^2 \tag{3}$$

Where, **n** is the total number of compounds with both experimental data and computational data, and **r** is the **common reference** in both experimental data and the relative binding free energy calculation.

Also,

$$\Delta\Delta G_{expt}^{r-i} = \Delta G_{expt}^{i} - \Delta G_{expt}^{r} \tag{3.1}$$

$$\Delta\Delta G_{ms\lambda d}^{r-i} = \Delta G_{ms\lambda d}^{i} - \Delta G_{ms\lambda d}^{r} \tag{3.2}$$

A is chosen to minimize the variance i.e., $\frac{d\sigma^2}{dA} = 0$. This yields **A** as,

$$A = \frac{\sum_{i=1}^{n}(\Delta\Delta G_{expt}^{r-i} - \Delta\Delta G_{ms\lambda d}^{r-i})}{n} \tag{4}$$

Finally, we compute the absolute binding affinities as:

$$\Delta G_{ms\lambda d}^{i} = \Delta G_{expt}^{r} + \Delta\Delta G_{ms\lambda d}^{r-i} + A \tag{5}$$

substituting **A** from equation 4 into equation 5:

$$\Delta G_{ms\lambda d}^{i} = \Delta G_{expt}^{r} + \Delta\Delta G_{ms\lambda d}^{r-i} + \frac{\sum_{i=1}^{n}(\Delta\Delta G_{expt}^{r-i} - \Delta\Delta G_{ms\lambda d}^{r-i})}{n} \tag{5.1}$$

$$\Delta G_{ms\lambda d}^{i} = \Delta G_{expt}^{r} + \Delta\Delta G_{ms\lambda d}^{r-i} + \frac{\sum_{i=1}^{n}(\Delta G_{expt}^{i} - \Delta G_{expt}^{r})}{n} - \frac{\sum_{i=1}^{n}\Delta\Delta G_{ms\lambda d}^{r-i}}{n} \tag{5.2}$$

$$\Delta G_{ms\lambda d}^{i} = \Delta G_{expt}^{r} + \Delta\Delta G_{ms\lambda d}^{r-i} + \frac{\sum_{i=1}^{n}\Delta G_{expt}^{i}}{n} - \frac{\sum_{i=1}^{n}\Delta G_{expt}^{r}}{n} - \frac{\sum_{i=1}^{n}\Delta\Delta G_{ms\lambda d}^{r-i}}{n} \tag{5.3}$$

We can write $(\frac{\sum_{i=1}^{n}\Delta G_{expt}^{r}}{n})$ as $(\frac{n\,\Delta G_{expt}^{r}}{n})$; substituting this in the above equation, we get:

$$\Delta G_{ms\lambda d}^{i} = \Delta G_{expt}^{r} + \Delta\Delta G_{ms\lambda d}^{r-i} + \frac{\sum_{i=1}^{n}\Delta G_{expt}^{i}}{n} - \frac{n\,\Delta G_{expt}^{r}}{n} - \frac{\sum_{i=1}^{n}\Delta\Delta G_{ms\lambda d}^{r-i}}{n} \tag{5.4}$$

Or we write it as,

$$\Delta G_{ms\lambda d}^{FF} = \Delta\Delta G_{ms\lambda d}^{FF} - \left(\frac{\sum \Delta\Delta G_{ms\lambda d}^{FF}}{n} - \frac{\sum \Delta\, G_{expt}}{n}\right) \tag{6}$$

PR3 holo-like structure modeling. The high structural similarity between PR3 and HNE and the scarcity of structural data for PR3 prompted us to use the structure of HNE complexed with compound 1 as a template. We overlayed the PR3 X-ray structure on the HNE X-ray structure docked with compound **1** in PyMOL. The resulting complex was saved as a PDB file containing PR3 and compound **1.** This complex, including crystal waters, was minimized, solvated in a cubic box (dimension: 67.6**4** Å) with TIP3P water molecules, neutralized with 0.15 M KCl salt ions, further minimized employing PBC and PME for long range electrostatics, heated gradually from 198 K to 298 K with increments of 1 K every 100 steps, and equilibrated in the NVT ensemble using Langevin dynamics for 60 ns with OpenMM (friction coefficient: 10 ps^{-1}) in CHARMM 45a2. The final structure of PR3 from MD was further used in the MSλD simulations.

I.2. Computational results

Table S2. The relative binding free energy predictions for HNE from the four different force field combinations calculated using equations 1-2.

Compound	$\Delta\Delta G^{FF}_{\mathrm{ms\lambda d}}$ (kcal/mol)			
	CHARMM-CGenFF	AMBER-GAFF2	OPLS-OPLS	CHARMM-OPLS
1	0.0 ± 0.1	0.0 ± 0.2	0.0 ± 0.4	0.0 ± 0.2
2	3.9 ± 0.2	3.5 ± 0.3	1.3 ± 0.4	3.3 ± 0.2
3	3.9 ± 0.1	1.9 ± 0.3	2.3 ± 0.4	2.1 ± 0.2
4	5.0 ± 0.1	3.6 ± 0.4	3.0 ± 0.2	4.0 ± 0.2
5	3.2 ± 0.1	0.5 ± 0.3	2.2 ± 0.2	1.8 ± 0.2
6	4.5 ± 0.2	1.0 ± 0.3	0.8 ± 0.2	1.1 ± 0.1
7	3.4 ± 0.1	-0.6 ± 0.3	-1.0 ± 0.2	0.7 ± 0.1
8	3.8 ± 0.1	0.6 ± 0.3	0.4 ± 0.3	1.1 ± 0.1
9	3.5 ± 0.1	1.1 ± 0.3	2.4 ± 0.4	2.9 ± 0.2
10	3.6 ± 0.1	2.3 ± 0.3	3.0 ± 0.2	2.8 ± 0.1
11	6.0 ± 0.2	2.5 ± 0.3	-0.1 ± 0.2	0.7 ± 0.1

Table S3. Comparison of predicted absolute free energies from MSλD against experimental data reported by Nussbaum et al.[42–44] ($\Delta G = RT\ln(IC_{50})$) for HNE. The predicted values were obtained with four different combinations of force fields for protein and ligand using data in **Table S2** and equation **6** with data from Nussbaum et al. for minimization of systematic errors.

Compound	ΔG_{expt}	$\Delta G^{FF}_{ms\lambda d}$ (kcal/mol)			
	kcal/mol	CHARMM-CGenFF	AMBER-GAFF2	OPLS-OPLS	CHARMM-OPLS
1 (22)	-13.8	-15.6 ± 0.1	-13.4 ± 0.2	-13.2 ± 0.4	-13.8 ± 0.2
2 (46)	-10.6	-11.7 ± 0.2	-9.9 ± 0.3	-11.9 ± 0.4	-10.5 ± 0.2
3 (48)	-11.7	-11.7 ± 0.1	-11.5 ± 0.3	-10.9 ± 0.4	-11.6 ± 0.2
4 (49)	-9.7	-10.6 ± 0.1	-9.8 ± 0.4	-10.2 ± 0.2	-9.8 ± 0.2
5 (50)	-12.5	-12.4 ± 0.1	-12.8 ± 0.3	-11.0 ± 0.2	-11.9 ± 0.2
6 (51)	-12.8	-11.1 ± 0.2	-12.3 ± 0.3	-12.4 ± 0.2	-12.6 ± 0.1
7 (53)	-13.3	-12.2 ± 0.1	-14.0 ± 0.3	-14.2 ± 0.2	-13.0 ± 0.1
8 (54)	-12.9	-11.9 ± 0.1	-12.8 ± 0.3	-12.8 ± 0.3	-12.7 ± 0.1
9 (55)	-10.8	-12.1 ± 0.1	-12.3 ± 0.3	-10.8 ± 0.4	-10.9 ± 0.2
10 (56)	-9.9	-12.0 ± 0.1	-11.1 ± 0.3	-10.2 ± 0.2	-10.9 ± 0.1
11 (58)	-12.9	-9.7 ± 0.2	-10.9 ± 0.3	-13.3 ± 0.2	-13.1 ± 0.1
RMSE	-	1.6	0.9	0.8	0.4
Pearson R	-	0.4	0.8	0.8	0.9
Spearman ρ	-	0.4	0.8	0.9	0.9

Table S4. Relative binding free energy predictions for PR3 from the four different combinations of force fields (equations **1**-**2**).

Compound	$\Delta\Delta G^{FF}_{ms\lambda d}$ (kcal/mol)			
	CHARMM-CGenFF	AMBER-GAFF2	OPLS-OPLS	CHARMM-OPLS
1	0.0 ± 0.3	0.0 ± 0.3	0.0 ± 0.7	0.0 ± 0.3
2	0.8 ± 0.2	1.1 ± 0.3	1.8 ± 0.3	2.0 ± 0.3
3	0.8 ± 0.2	-0.2 ± 0.2	2.3 ± 0.5	0.0 ± 0.3
4	1.5 ± 0.2	1.5 ± 0.2	3.5 ± 0.3	2.6 ± 0.3
5	0.5 ± 0.2	-1.5 ± 0.2	2.5 ± 0.3	0.7 ± 0.3
6	-0.1 ± 0.2	-0.7 ± 0.2	1.9 ± 0.3	0.8 ± 0.3
7	-1.3 ± 0.2	-2.8 ± 0.2	1.1 ± 0.3	-0.6 ± 0.3
8	-1.3 ± 0.2	-1.7 ± 0.2	1.5 ± 0.3	0.0 ± 0.3
9	-0.2 ± 0.3	-1.5 ± 0.3	4.6 ± 0.4	3.2 ± 0.3
10	-0.4 ± 0.3	0.2 ± 0.3	3.5 ± 0.5	2.7 ± 0.3
11	0.9 ± 0.3	0.2 ± 0.3	1.9 ± 0.3	1.2 ± 0.3

Table S5. Absolute predicted binding free energy for PR3 from the four different combinations of force fields. The absolute values are obtained from the data in **Table S4** and experimental IC_{50} for compound **1** (**Table 3**) using equation **6**.

Compound	$\Delta G^{FF}_{ms\lambda d}$ (kcal/mol)			
	CHARMM-CGenFF	AMBER-GAFF2	OPLS-OPLS	CHARMM-OPLS
1	-9.5 ± 0.3	-9.5 ± 0.3	-9.5 ± 0.7	-9.5 ± 0.3
2	-8.7 ± 0.2	-8.4 ± 0.3	-7.7 ± 0.3	-7.5 ± 0.3
3	-8.7 ± 0.2	-9.7 ± 0.2	-7.2 ± 0.5	-9.5 ± 0.3
4	-8.0 ± 0.2	-8.0 ± 0.2	-6.0 ± 0.3	-6.9 ± 0.3
5	-9.0 ± 0.2	-11.0 ± 0.2	-7.0 ± 0.3	-8.8 ± 0.3
6	-9.6 ± 0.2	-10.2 ± 0.2	-7.6 ± 0.3	-8.7 ± 0.3
7	-10.8 ± 0.2	-12.3 ± 0.2	-8.4 ± 0.3	-10.1 ± 0.3
8	-10.8 ± 0.2	-11.2 ± 0.2	-8.0 ± 0.3	-9.5 ± 0.3
9	-9.7 ± 0.3	-11.0 ± 0.3	-4.9 ± 0.4	-6.3 ± 0.3
10	-9.9 ± 0.3	-9.3 ± 0.3	-6.0 ± 0.5	-6.8 ± 0.3
11	-8.6 ± 0.3	-9.3 ± 0.3	-7.6 ± 0.3	-8.3 ± 0.3

Table S6. Absolute predicted binding free energy predictions for HNE from the four different combinations of force fields; values are calculated from ΔΔG data in **Table S2** using equation **6** and only the experimental data for compound **1** (**Table 3**) as an anchor consistent with PR3 data in **Table S5**.

Compound	$\Delta G^{FF}_{ms\lambda d}$ (kcal/mol)			
	CHARMM-CGenFF	AMBER-GAFF2	OPLS-OPLS	CHARMM-OPLS
1	-12.6 ± 0.1	-12.6 ± 0.2	-12.6 ± 0.4	-12.6 ± 0.2
2	-8.7 ± 0.2	-9.1 ± 0.3	-11.3 ± 0.4	-9.3 ± 0.2
3	-8.7 ± 0.1	-10.7 ± 0.3	-10.3 ± 0.4	-10.5 ± 0.2
4	-7.6 ± 0.1	-9.0 ± 0.4	-9.6 ± 0.2	-8.6 ± 0.2
5	-9.4 ± 0.1	-12.1 ± 0.3	-10.4 ± 0.2	-10.8 ± 0.2
6	-8.1 ± 0.2	-11.6 ± 0.3	-11.8 ± 0.2	-11.5 ± 0.1
7	-9.2 ± 0.1	-13.2 ± 0.3	-13.6 ± 0.2	-11.9 ± 0.1
8	-8.8 ± 0.1	-12.0 ± 0.3	-12.2 ± 0.3	-11.5 ± 0.1
9	-9.1 ± 0.1	-11.5 ± 0.3	-10.2 ± 0.4	-9.7 ± 0.2
10	-9.0 ± 0.1	-10.3 ± 0.3	-9.6 ± 0.2	-9.8 ± 0.1
11	-6.6 ± 0.2	-10.1 ± 0.3	-12.7 ± 0.2	-11.9 ± 0.1

Table S7. Predicted IC_{50} and pIC_{50} values for Bayer compounds **1-11** towards PR3. The IC_{50} (and pIC_{50}) values are calculated from the predicted binding free energies in **Table S5** ($IC_{50} = \exp(\Delta G/RT)$).

Compound	IC_{50} (nM)	pIC_{50}
1	102.2	7.0
2	3027.8	5.5
3	102.2	7.0
4	8368.4	5.1
5	334.6	6.5
6	396.4	6.4
7	37.0	7.4
8	102.2	7.0
9	23128.7	4.6
10	9913.5	5.0
11	780.6	6.1

Table S8. Comparison of the MSλD predicted binding free energies with four different combinations of force fields and experimentally determined potency for PR3. Absolute values are obtained from the relative binding free energy data of compound 1 and compounds 5-8 (Table S4) and their corresponding experimental data using equation 6.

Compound	Expt[a]	$\Delta G_{ms\lambda d}^{FF}$ (kcal/mol)			
		CHARMM-CGenFF	AMBER-GAFF2	OPLS-OPLS	CHARMM-OPLS
1	-9.5	-9.6 ± 0.3	-8.7 ± 0.3	-11.4 ± 0.7	-10.2 ± 0.3
5	-9.1	-9.1 ± 0.2	-10.2 ± 0.2	-8.9 ± 0.3	-9.5 ± 0.3
6	-11.0	-9.7 ± 0.2	-9.3 ± 0.2	-9.5 ± 0.3	-9.4 ± 0.3
7	-10.4	-10.9 ± 0.2	-11.4 ± 0.2	-10.3 ± 0.3	-10.8 ± 0.3
8	-9.8	-10.9 ± 0.2	-10.3 ± 0.2	-9.9 ± 0.3	-10.2 ± 0.3
RMSE	-	0.8	1.1	1.1	0.8

[a] The uncertainty(σ) in ΔG_{expt} is at most 0.1 kcal/mol. The uncertainty in ΔG_{expt} is calculated from uncertainty in the corresponding IC_{50} value as: $\sigma_{\Delta G_{expt}} = \left|\frac{d\Delta G_{expt}}{dIC_{50}}\right| * \sigma_{IC_{50}} = \left|\frac{RT}{IC_{50}}\right| * \sigma_{IC_{50}}$; where $\Delta G_{expt} = RT\ln(IC_{50})$ and both IC_{50} and $\sigma_{IC_{50}}$ should be in M (mol/L).

Table S9. Absolute predicted binding free energy predictions for HNE from CHARMM-OPLS FF combination; values are calculated from ΔΔG data in **Table S2** using equation 5 and all *our* experimental data points for both HNE and PR3 to estimate the shift for minimization of systematic errors. Mean unsigned error (MUE) is only calculated using the data points with corresponding experimental data.

Compound	ΔG_{expt}	$\Delta G_{ms\lambda d}^{CHARMM-OPLS}$
	(kcal/mol)	
1	-12.6	-13.3 ± 0.2
2		-10.0 ± 0.2
3		-11.2 ± 0.2
4		-9.3 ± 0.2
5	-12.1	-11.5 ± 0.2
6	-12.5	-12.2 ± 0.1
7	-12.2	-12.6 ± 0.1
8	-12.4	-12.2 ± 0.1
9		-10.4 ± 0.2
10		-10.5 ± 0.1
11		-12.6 ± 0.1
MUE		0.43

Table S10. Absolute predicted binding free energy values for PR3 from CHARMM-OPLS FF combination; values are calculated from ΔΔG data in **Table S4** using equation 5 and all our experimental data points for both HNE and PR3 to estimate the shift for minimization of systematic errors. Mean unsigned error (MUE) is only calculated using the data points with corresponding experimental data (ΔG_{expt}).

Compound	ΔG_{expt}	$\Delta G_{ms\lambda d}^{CHARMM-OPLS}$
	(kcal/mol)	
1	-9.5	-10.2 ± 0.3
2		-8.2 ± 0.3
3		-10.2 ± 0.3
4		-7.6 ± 0.3
5	-9.1	-9.5 ± 0.3
6	-11.0	-9.4 ± 0.3
7	-10.4	-10.8 ± 0.3
8	-9.8	-10.2 ± 0.3
9		-7.0 ± 0.3
10		-7.5 ± 0.3
11		-9.0 ± 0.3
MUE		0.66

II. Enzymatic assays

II.1 Experimental details

Chemicals. All chemicals and reagents were of analytical grade. Enzymes, PR3 and HNE, were purchased from Athens Research and Technology. Compound **1** was purchased from Medchem Express while compounds **5-8** were prepared by Syngene International Limited (Bangalore, India) using published procedures.[42] The substrate of HNE (MeOSuc-AAPV-AMC) was supplied by Santa Cruz Biotechnology.

Activity assay. The enzymatic assays for both PR3 and HNE were conducted according to the method published in (Budnjo et al., 2014) with minor modifications. Briefly, 0.5 nM of enzyme was incubated with buffer (50 mM HEPES pH 7.4, 750 mM NaCl, 0.5 % Igepal and 1 % DMSO) or same buffer containing the inhibitors at various concentrations for 30 min at RT. For PR3, the reaction was initiated by adding the FRET peptide (Abz-VADnVADYQ-EDDnp, excitation filter 320, emission filter 420) at a final concentration of 5 μM while the HNE reaction was started by adding the fluorogenic substrate (MeOSuc-AAPV-AMC, excitation filter 360, emission filter 460) at a final concentration of 5 μM. The fluorescent signal was read every 30 second for 30 min at 30 °C using a fluorescent plate reader (Tecan Spark). The enzymatic activity was measured from the initial linear portion of the slope (fluorescent signal/min) of the time-course curve of reaction progress. The activity data were produced in triplicates. Data were fitted using nonlinear regression analysis (GraphPad Prism v.9) to determine the IC_{50} values.

II.2. Results

Table S11. Experimentally determined potency for the racemates (±) and the S-enantiomers.

Compound	IC_{50} (nM)	
	HNE	PR3
(±)-**5**	2.3 ± 0.6	331.0 ± 84.3
(*S*)-**5**	128.2 ± 14.2	29300 ± 1300
(±)-**6**	1.4 ± 0.1	16.3 ± 0.1
(*S*)-**6**	798.2 ± 42.6	19500 ± 3100
(±)-**7**	1.4 ± 0.1	36.3 ± 3.1
(*S*)-**7**	84.4 ± 16.6	4760 ± 426.6
(±)-**8**	1.5 ± 0.3	133.3 ± 14.4
(*S*)-**8**	122.1± 34.3	6300 ± 770.0

Figure S1: Dose-response curves for the studied inhibitors with both PR3 and HNE. All activity measurements for each concentration of compounds were conducted in triplicates. For a given inhibitor, each data point on the curve represents the average of two to three replicates and error bars represent the standard deviation. The non-linear fit is performed using GraphPad Prism® and a non-linear fit with a standard slope (Hill slope = - 1). This procedure takes the error on each "average" point into account, and we report the corresponding 95% confidence interval (95% CI) below each graph.

a

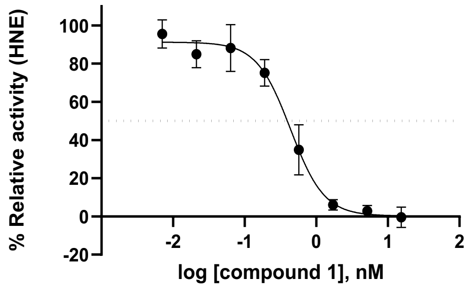

95% CI: 0.3383 to 0.5584

b

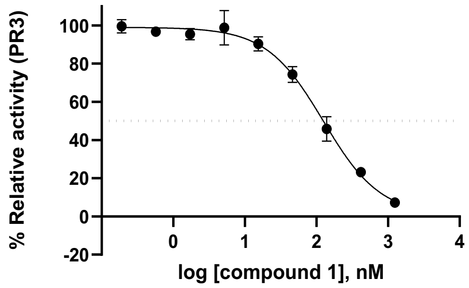

95% CI: 97.68 to 175.6

c

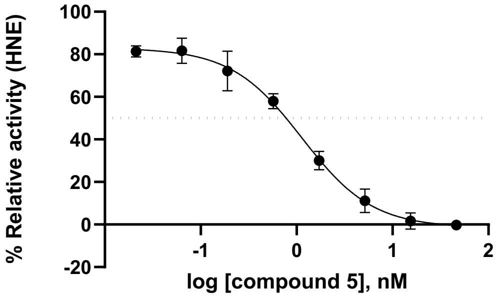

95% CI: 0.8718 to 1.472

d

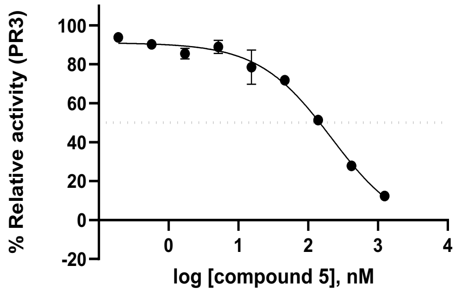

95% CI: 137.9 to 573.4

e

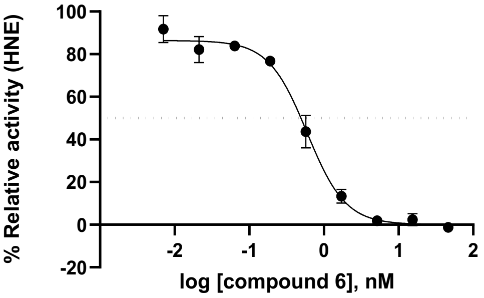

95% CI: 0.5081 to 0.6953

f

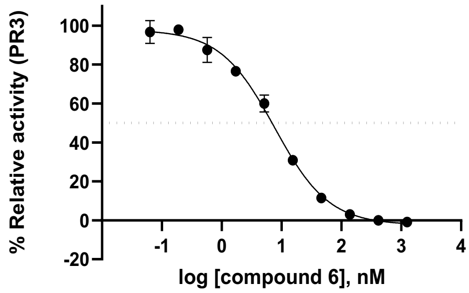

95% CI: 6.484 to 8.954

g

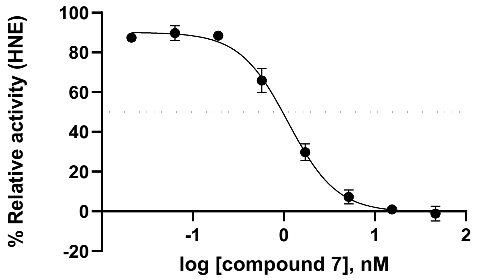

95% CI: 0.9777 to 1.269

h

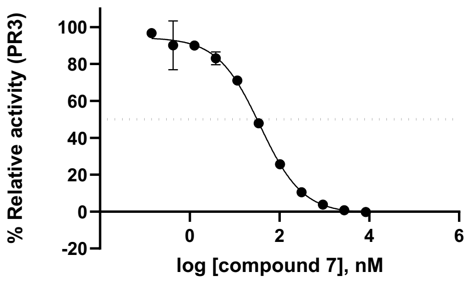

95% CI: 30.28 to 44.62

i

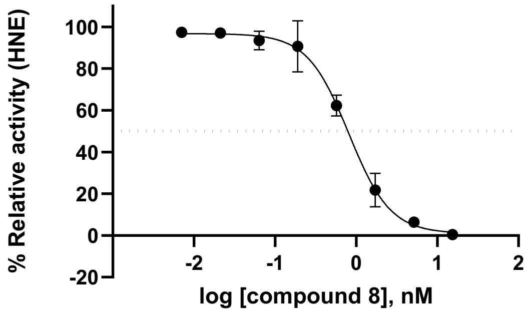

95% CI: 0.6777 to 0.9822

j

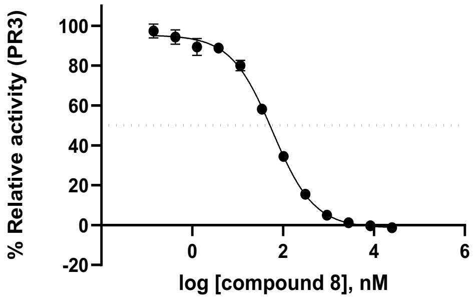

95% CI: 52.59 to 65.68

III. Compounds synthesis

General considerations for the synthesis of compound 5-8

Compounds **5-8** were prepared as racemic mixtures following published protocols (Scheme 1).[42] Each of the enantiomers of **5-8** were isolated following purification by supercritical fluid chromatography (SFC) on a chiral column as described below. The absolute configuration of the isolated materials was assigned based on the measured inhibitory activity against HNE (see above).

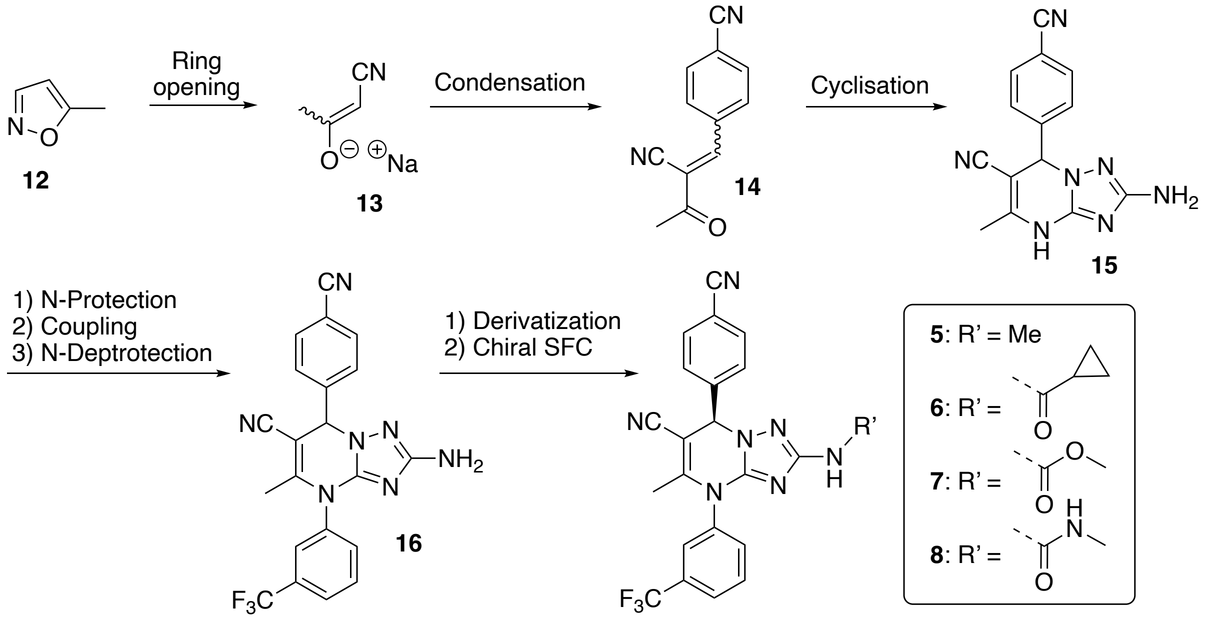

Scheme 1: Synthesis protocol for compounds **5-8**.

(*R*)-7-(4-cyanophenyl)-5-methyl-2-(methylamino)-4-(3-(trifluoromethyl)phenyl)-4,7-dihydro-[1,2,4]triazolo[1,5-*a*]pyrimidine-6-carbonitrile (5)

Racemic compound **5** was prepared as previously reported. The racemic mixture was separated using SFC chromatography on a Chiralpak IG column eluting with 20% methanol as co-solvent. R_t = 3.13 (S-isomer), R_t = 4.08 (R-isomer).

^{1}H NMR (400 MHz, $(CD_3)_2SO$): δ = 8.14 (s, 1H), 7.99 – 7.86 (m, 4H), 7.80 (t, *J* = 7.8, 1H), 7.71 (d, *J* = 8.0, 2H), 6.24 (d, *J* = 1.3, 1H), 6.00 (q, *J* = 5.0, 1H), 2.47 (d, *J* = 5.0, 3H), 1.96 (d, *J* = 1.0, 3H).
Purity: 96.4% (LC-MS), 91% ee (Chiralpak IG, 254 nm).

(*R*)-*N*-(6-cyano-7-(4-cyanophenyl)-5-methyl-4-(3-(trifluoromethyl)phenyl)-4,7-dihydro-[1,2,4]triazolo[1,5-*a*]pyrimidin-2-yl)cyclopropanecarboxamide (6)

Racemic compound **6** was prepared as previously reported. The racemic mixture was separated using SFC chromatography on a YMC Cellulose-SC column eluting with 50% methanol as co-solvent. R_t = 1.39 (R-isomer), R_t = 4.23 (S-isomer).

^{1}H NMR (400 MHz, $(CD_3)_2SO$): δ = 10.72 (s, 1H), 8.21 (s, 1H), 7.95 (dd, *J* = 17.2, 8.1, 4H), 7.87 – 7.66 (m, 3H), 6.43 (d, *J* = 1.3, 1H), 1.99 (d, *J* = 1.1, 3H), 1.69 (s, 1H), 0.70-0.63 (m, 4H).
Purity: 99.2% (LC-MS), 100% ee (YMC Cellulose-SC, 254 nm).

Methyl (*R*)-(6-cyano-7-(4-cyanophenyl)-5-methyl-4-(3-(trifluoromethyl)phenyl)-4,7-dihydro-[1,2,4]triazolo[1,5-*a*]pyrimidin-2-yl)carbamate (7)

Racemic compound **7** was prepared as previously reported. The racemic mixture was separated using SFC chromatography on a YMC Cellulose-SC column eluting with 50% methanol as co-solvent. R_t = 1.69 (R-isomer), R_t = 4.40 (S-isomer).

^{1}H NMR (400 MHz, $(CD_3)_2SO$) δ = 10.07 (s, 1H), 8.20 (s, 1H), 8.04 – 7.86 (m, 4H), 7.86 – 7.68 (m, 3H), 6.44 (d, *J* = 1.3, 1H), 3.51 (s, 3H), 1.97 (d, *J* = 1.1, 3H).
Purity: 99.2% (LC-MS), 100% ee (YMC Cellulose-SC, 254 nm).

(*R*)-1-(6-cyano-7-(4-cyanophenyl)-5-methyl-4-(3-(trifluoromethyl)phenyl)-4,7-dihydro-[1,2,4]triazolo[1,5-*a*]pyrimidin-2-yl)-3-methylurea (8)

Racemic compound **8** was prepared as previously reported. The racemic mixture was separated using SFC chromatography on a Chiralpak IG column eluting with 50% methanol as co-solvent. R_t = 1.90 (S-isomer), R_t = 3.04 (R-isomer

^{1}H NMR (400 MHz, $(CD_3)_2SO$): δ = 9.42 (s, 1H), 8.20 (s, 1H), 8.05 – 7.88 (m, 4H), 7.88 – 7.70 (m, 3H), 6.95 (d, *J* = 4.6, 1H), 6.40 (d, *J* = 1.2, 1H), 2.00 (d, *J* = 1.0, 3H).
Purity: 99.1% (LC-MS), 99.3% ee (Chiralpak IG, 254 nm).

Figure S2: Chromatograms from chiral SFC analysis of isolated enantiomers of **5**-**8**.

a

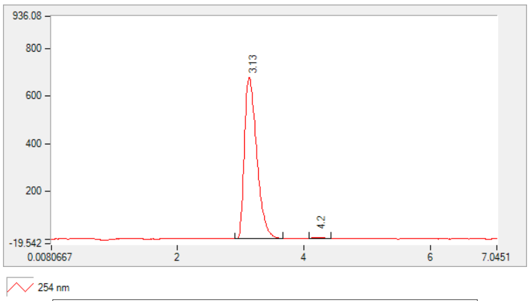

Results UV3			
Sr.No.	RT	Area	Area %
1	3.13	9142.025	99.487
2	4.20	47.184	0.513

S-enantiomer of compound **5**

b

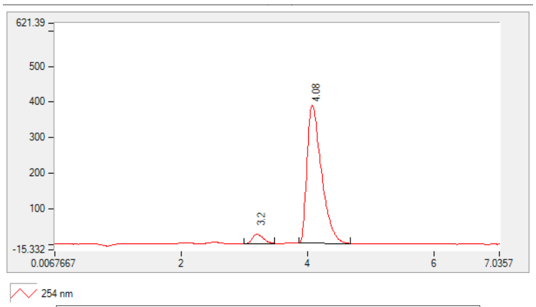

Results UV3			
Sr.No.	RT	Area	Area %
1	3.20	301.625	4.619
2	4.08	6227.992	95.381

Compound **5**

c

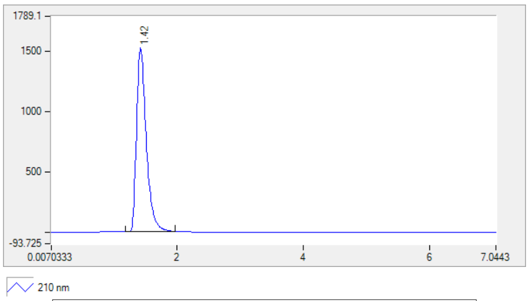

Results UV1			
Sr.No.	RT	Area	Area %
1	1.42	16287.344	100.000

Compound **6**

d

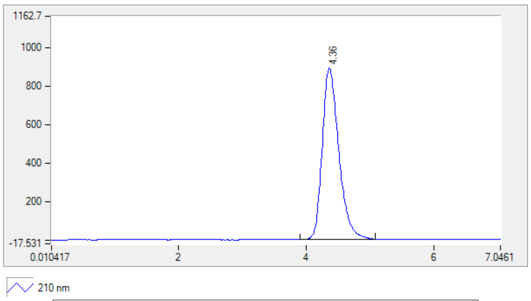

Results UV1			
Sr.No.	RT	Area	Area %
1	4.36	15928.172	100.000

S-enantiomer of compound **6**

e

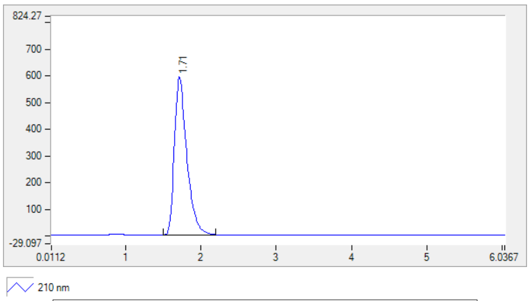

Results UV1			
Sr.No.	RT	Area	Area %
1	1.71	6791.209	100.000

Compound **7**

f

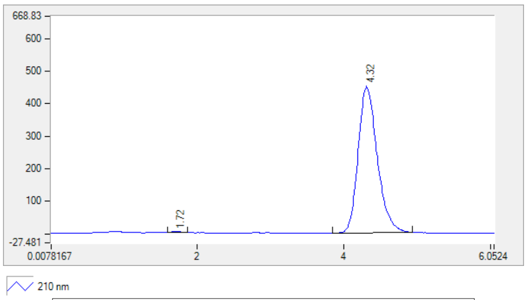

Results UV1			
Sr.No.	RT	Area	Area %
1	1.72	30.462	0.377
2	4.32	8040.875	99.623

S-enantiomer of compound **7**

g

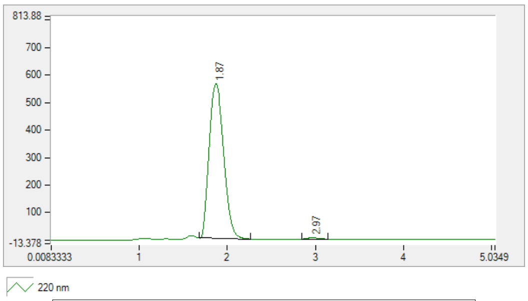

Results UV2			
Sr.No.	RT	Area	Area %
1	1.87	6605.233	99.265
2	2.97	48.916	0.735

S-enantiomer of compound **8**

h

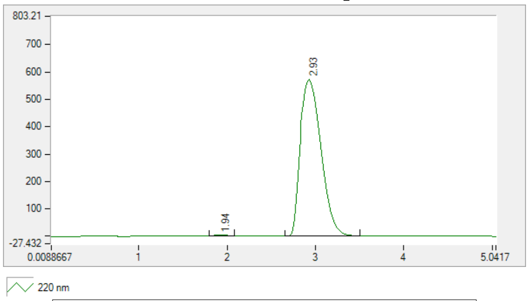

Results UV2			
Sr.No.	RT	Area	Area %
1	1.94	31.733	0.337
2	2.93	9391.000	99.663

Compound **8**

References

(1) Warren DeLano; Schrodinger LLC. The PyMOL Molecular Graphic System.

(2) Šali, A.; Blundell, T. L. Comparative Protein Modelling by Satisfaction of Spatial Restraints. *J. Mol. Biol.* **1993**, *234*, 779–815. https://doi.org/10.1006/jmbi.1993.1626.

(3) Yang, Z.; Lasker, K.; Schneidman-Duhovny, D.; Webb, B.; Huang, C. C.; Pettersen, E. F.; Goddard, T. D.; Meng, E. C.; Sali, A.; Ferrin, T. E. UCSF Chimera, MODELLER, and IMP: An Integrated Modeling System. *J. Struct. Biol.* **2012**, *179*, 269–278. https://doi.org/10.1016/j.jsb.2011.09.006.

(4) Hajjar, E.; Korkmaz, B.; Gauthier, F.; Brandsdal, B. O.; Witko-Sarsat, V.; Reuter, N. Inspection of the Binding Sites of Proteinase3 for the Design of a Highly Specific Substrate. *J. Med. Chem.* **2006**, *49*, 1248–1260. https://doi.org/10.1021/jm051018t.

(5) Li, H.; Robertson, A. D.; Jensen, J. H. Very Fast Empirical Prediction and Rationalization of Protein pK $_a$ Values. *Proteins Struct. Funct. Bioinforma.* **2005**, *61*, 704–721. https://doi.org/10.1002/prot.20660.

(6) Hajjar, E.; Dejaegere, A.; Reuter, N. Challenges in p *K* $_a$ Predictions for Proteins: The Case of Asp213 in Human Proteinase 3. *J. Phys. Chem. A* **2009**, *113*, 11783–11792. https://doi.org/10.1021/jp902930u.

(7) Fujinaga, M.; Chernaia, M. M.; Halenbeck, R.; Koths, K.; James, M. N. G. The Crystal Structure of PR3, a Neutrophil Serine Proteinase Antigen of Wegener's Granulomatosis Antibodies. *J. Mol. Biol.* **1996**, *261*, 267–278. https://doi.org/10.1006/jmbi.1996.0458.

(8) Chemaxon. MarvinSketch, 2020.

(9) O'Boyle, N. M.; Banck, M.; James, C. A.; Morley, C.; Vandermeersch, T.; Hutchison, G. R. Open Babel: An Open Chemical Toolbox. *J. Cheminformatics* **2011**, *3*, 33. https://doi.org/10.1186/1758-2946-3-33.

(10) Trott, O.; Olson, A. J. AutoDock Vina: Improving the Speed and Accuracy of Docking with a New Scoring Function, Efficient Optimization, and Multithreading. *J. Comput. Chem.* **2009**, NA-NA. https://doi.org/10.1002/jcc.21334.

(11) Vanommeslaeghe, K.; MacKerell, A. D. Automation of the CHARMM General Force Field (CGenFF) I: Bond Perception and Atom Typing. *J. Chem. Inf. Model.* **2012**, *52*, 3144–3154. https://doi.org/10.1021/ci300363c.

(12) Vanommeslaeghe, K.; Raman, E. P.; MacKerell, A. D. Automation of the CHARMM General Force Field (CGenFF) II: Assignment of Bonded Parameters and Partial Atomic Charges. *J. Chem. Inf. Model.* **2012**, *52*, 3155–3168. https://doi.org/10.1021/ci3003649.

(13) Vanommeslaeghe, K.; Hatcher, E.; Acharya, C.; Kundu, S.; Zhong, S.; Shim, J.; Darian, E.; Guvench, O.; Lopes, P.; Vorobyov, I.; Mackerell, A. D. CHARMM General Force Field: A Force Field for Drug-like Molecules Compatible with the CHARMM All-Atom Additive Biological Force Fields. *J. Comput. Chem.* **2009**, NA-NA. https://doi.org/10.1002/jcc.21367.

(14) Yu, W.; He, X.; Vanommeslaeghe, K.; MacKerell, A. D. Extension of the CHARMM General Force Field to Sulfonyl-Containing Compounds and Its Utility in Biomolecular Simulations. *J. Comput. Chem.* **2012**, *33*, 2451–2468. https://doi.org/10.1002/jcc.23067.

(15) Case, D. A.; Aktulga, H. M.; Belfon, K.; Ben-Shalom, I. Y.; Berryman, J. T.; Brozell, S. R.; Cerutti, D. S.; Cheatham III, T. E.; Cisneros, G. A.; Cruzeiro, V. W. D.; Darden, T. A.; Forouzesh, N.; Giambaşu, G.; Giese, T.; Gilson, M. K.; Gohlke, H.; Goetz, A. W.; Harris,

J.; Izadi, S.; Izmailov, S. A.; Kasavajhala, K.; Kaymak, M. C.; King, E.; Kovalenko, A.; Kurtzman, T.; Lee, T. S.; Li, P.; Lin, C.; Liu, J.; Luchko, T.; Luo, R.; Machado, M.; Man, V.; Manathunga, M.; Merz, K. M.; Miao, Y.; Mikhailovskii, O.; Monard, G.; Nguyen, H.; O'Hearn, K. A.; Onufriev, A.; Pan, F.; Pantano, S.; Qi, R.; Rahnamoun, A.; Roe, D. R.; Roitberg, A.; Sagui, C.; Schott-Verdugo, S.; Shajan, A.; Shen, J.; Simmerling, C. L.; Skrynnikov, N. R.; Smith, J.; Swails, J.; Walker, R. C.; Wang, J.; Wang, J.; Wei, H.; Wu, X.; Wu, Y.; Xiong, Y.; Xue, Y.; York, D. M.; Zhao, S.; Zhu, Q.; Kollman, P. A. Amber 2022, 2022.

(16) Wang, J.; Wang, W.; Kollman, P. A.; Case, D. A. Automatic Atom Type and Bond Type Perception in Molecular Mechanical Calculations. *J. Mol. Graph. Model.* **2006**, *25*, 247–260. https://doi.org/10.1016/j.jmgm.2005.12.005.

(17) He, X.; Man, V. H.; Yang, W.; Lee, T.-S.; Wang, J. A Fast and High-Quality Charge Model for the next Generation General AMBER Force Field. *J. Chem. Phys.* **2020**, *153*, 114502. https://doi.org/10.1063/5.0019056.

(18) Jorgensen, W. L.; Tirado-Rives, J. Potential Energy Functions for Atomic-Level Simulations of Water and Organic and Biomolecular Systems. *Proc. Natl. Acad. Sci.* **2005**, *102*, 6665–6670. https://doi.org/10.1073/pnas.0408037102.

(19) Dodda, L. S.; Vilseck, J. Z.; Tirado-Rives, J.; Jorgensen, W. L. 1.14*CM1A-LBCC: Localized Bond-Charge Corrected CM1A Charges for Condensed-Phase Simulations. *J. Phys. Chem. B* **2017**, *121*, 3864–3870. https://doi.org/10.1021/acs.jpcb.7b00272.

(20) Dodda, L. S.; Cabeza de Vaca, I.; Tirado-Rives, J.; Jorgensen, W. L. LigParGen Web Server: An Automatic OPLS-AA Parameter Generator for Organic Ligands. *Nucleic Acids Res.* **2017**, *45*, W331–W336. https://doi.org/10.1093/nar/gkx312.

(21) Jorgensen, W. L.; Tirado-Rives, J. Molecular Modeling of Organic and Biomolecular Systems usingBOSS andMCPRO. *J. Comput. Chem.* **2005**, *26*, 1689–1700. https://doi.org/10.1002/jcc.20297.

(22) Jorgensen, W. L.; Tirado-Rives, J. The OPLS [Optimized Potentials for Liquid Simulations] Potential Functions for Proteins, Energy Minimizations for Crystals of Cyclic Peptides and Crambin. *J. Am. Chem. Soc.* **1988**, *110*, 1657–1666. https://doi.org/10.1021/ja00214a001.

(23) Jorgensen, W. L.; Maxwell, D. S.; Tirado-Rives, J. Development and Testing of the OPLS All-Atom Force Field on Conformational Energetics and Properties of Organic Liquids. *J. Am. Chem. Soc.* **1996**, *118*, 11225–11236. https://doi.org/10.1021/ja9621760.

(24) Pettersen, E. F.; Goddard, T. D.; Huang, C. C.; Couch, G. S.; Greenblatt, D. M.; Meng, E. C.; Ferrin, T. E. UCSF Chimera?A Visualization System for Exploratory Research and Analysis. *J. Comput. Chem.* **2004**, *25*, 1605–1612. https://doi.org/10.1002/jcc.20084.

(25) Vilseck, J. Z.; Cervantes, L. F.; Hayes, R. L.; Brooks, C. L. Optimizing Multisite λ-Dynamics Throughput with Charge Renormalization. *J. Chem. Inf. Model.* **2022**, acs.jcim.2c00047. https://doi.org/10.1021/acs.jcim.2c00047.

(26) Brooks, B. R.; Brooks, C. L.; Mackerell, A. D.; Nilsson, L.; Petrella, R. J.; Roux, B.; Won, Y.; Archontis, G.; Bartels, C.; Boresch, S.; Caflisch, A.; Caves, L.; Cui, Q.; Dinner, A. R.; Feig, M.; Fischer, S.; Gao, J.; Hodoscek, M.; Im, W.; Kuczera, K.; Lazaridis, T.; Ma, J.; Ovchinnikov, V.; Paci, E.; Pastor, R. W.; Post, C. B.; Pu, J. Z.; Schaefer, M.; Tidor, B.; Venable, R. M.; Woodcock, H. L.; Wu, X.; Yang, W.; York, D. M.; Karplus, M. CHARMM: The Biomolecular Simulation Program. *J. Comput. Chem.* **2009**, *30*, 1545–1614. https://doi.org/10.1002/jcc.21287.

(27) Brooks, B. R.; Bruccoleri, R. E.; Olafson, B. D.; States, D. J.; Swaminathan, S.; Karplus, M. CHARMM: A Program for Macromolecular Energy, Minimization, and Dynamics Calculations. *J. Comput. Chem.* **1983**, *4*, 187–217. https://doi.org/10.1002/jcc.540040211.

(28) Pearlman, D. A. A Comparison of Alternative Approaches to Free Energy Calculations. *J. Phys. Chem.* **1994**, *98*, 1487–1493. https://doi.org/10.1021/j100056a020.

(29) Boresch, S.; Karplus, M. The Role of Bonded Terms in Free Energy Simulations: 1. Theoretical Analysis. *J. Phys. Chem. A* **1999**, *103*, 103–118. https://doi.org/10.1021/jp981628n.

(30) Feig, M.; Karanicolas, J.; Brooks, C. L. MMTSB Tool Set: Enhanced Sampling and Multiscale Modeling Methods for Applications in Structural Biology. *J. Mol. Graph. Model.* **2004**, *22*, 377–395. https://doi.org/10.1016/j.jmgm.2003.12.005.

(31) Knight, J. L.; Brooks, C. L. Multisite λ Dynamics for Simulated Structure–Activity Relationship Studies. *J. Chem. Theory Comput.* **2011**, *7*, 2728–2739. https://doi.org/10.1021/ct200444f.

(32) Hayes, R. L.; Armacost, K. A.; Vilseck, J. Z.; Brooks, C. L. Adaptive Landscape Flattening Accelerates Sampling of Alchemical Space in Multisite λ Dynamics. *J. Phys. Chem. B* **2017**, *121*, 3626–3635. https://doi.org/10.1021/acs.jpcb.6b09656.

(33) Raman, E. P.; Paul, T. J.; Hayes, R. L.; Brooks, C. L. Automated, Accurate, and Scalable Relative Protein–Ligand Binding Free-Energy Calculations Using Lambda Dynamics. *J. Chem. Theory Comput.* **2020**, *16*, 7895–7914. https://doi.org/10.1021/acs.jctc.0c00830.

(34) Hayes, R. L.; Buckner, J.; Brooks, C. L. BLaDE: A Basic Lambda Dynamics Engine for GPU-Accelerated Molecular Dynamics Free Energy Calculations. *J. Chem. Theory Comput.* **2021**, *17*, 6799–6807. https://doi.org/10.1021/acs.jctc.1c00833.

(35) Feller, S. E.; Zhang, Y.; Pastor, R. W.; Brooks, B. R. Constant Pressure Molecular Dynamics Simulation: The Langevin Piston Method. *J. Chem. Phys.* **1995**, *103*, 4613–4621. https://doi.org/10.1063/1.470648.

(36) Miyamoto, S.; Kollman, P. A. Settle: An Analytical Version of the SHAKE and RATTLE Algorithm for Rigid Water Models. *J. Comput. Chem.* **1992**, *13*, 952–962. https://doi.org/10.1002/jcc.540130805.

(37) Kumar, S.; Rosenberg, J. M.; Bouzida, D.; Swendsen, R. H.; Kollman, P. A. THE Weighted Histogram Analysis Method for Free-Energy Calculations on Biomolecules. I. The Method. *J. Comput. Chem.* **1992**, *13*, 1011–1021. https://doi.org/10.1002/jcc.540130812.

(38) Kong, X.; Brooks, C. L. Λ-dynamics: A New Approach to Free Energy Calculations. *J. Chem. Phys.* **1996**, *105*, 2414–2423. https://doi.org/10.1063/1.472109.

(39) Shirts, M. R.; Chodera, J. D. Statistically Optimal Analysis of Samples from Multiple Equilibrium States. *J. Chem. Phys.* **2008**, *129*, 124105. https://doi.org/10.1063/1.2978177.

(40) Klimovich, P. V.; Shirts, M. R.; Mobley, D. L. Guidelines for the Analysis of Free Energy Calculations. *J. Comput. Aided Mol. Des.* **2015**, *29*, 397–411. https://doi.org/10.1007/s10822-015-9840-9.

(41) Wang, L.; Wu, Y.; Deng, Y.; Kim, B.; Pierce, L.; Krilov, G.; Lupyan, D.; Robinson, S.; Dahlgren, M. K.; Greenwood, J.; Romero, D. L.; Masse, C.; Knight, J. L.; Steinbrecher, T.; Beuming, T.; Damm, W.; Harder, E.; Sherman, W.; Brewer, M.; Wester, R.; Murcko, M.; Frye, L.; Farid, R.; Lin, T.; Mobley, D. L.; Jorgensen, W. L.; Berne, B. J.; Friesner, R. A.;

Abel, R. Accurate and Reliable Prediction of Relative Ligand Binding Potency in Prospective Drug Discovery by Way of a Modern Free-Energy Calculation Protocol and Force Field. *J. Am. Chem. Soc.* **2015**, *137*, 2695–2703. https://doi.org/10.1021/ja512751q.

(42) von Nussbaum, Franz; Li, V. M.; Meibom, D.; Anlauf, S.; Bechem, M.; Delbeck, M.; Gerisch, M.; Harrenga, A.; Karthaus, D.; Lang, D.; Lustig, K.; Mittendorf, J.; Schäfer, M.; Schäfer, S.; Schamberger, J. Potent and Selective Human Neutrophil Elastase Inhibitors with Novel Equatorial Ring Topology: In Vivo Efficacy of the Polar Pyrimidopyridazine BAY-8040 in a Pulmonary Arterial Hypertension Rat Model. *ChemMedChem* **2016**, *11*, 199–206. https://doi.org/10.1002/cmdc.201500269.

(43) von Nussbaum, F.; Li, V. M.-J.; Allerheiligen, S.; Anlauf, S.; Bärfacker, L.; Bechem, M.; Delbeck, M.; Fitzgerald, M. F.; Gerisch, M.; Gielen-Haertwig, H.; Haning, H.; Karthaus, D.; Lang, D.; Lustig, K.; Meibom, D.; Mittendorf, J.; Rosentreter, U.; Schäfer, M.; Schäfer, S.; Schamberger, J.; Telan, L. A.; Tersteegen, A. Freezing the Bioactive Conformation to Boost Potency: The Identification of BAY 85-8501, a Selective and Potent Inhibitor of Human Neutrophil Elastase for Pulmonary Diseases. *ChemMedChem* **2015**, *10*, 1163–1173. https://doi.org/10.1002/cmdc.201500131.

(44) von Nussbaum, Franz; Karthaus, Dagmar; Anlauf, Sonja; Delbeck, Martina; Min-Jian, Li Volkhart; Meibom, Daniel; Lustig, Klemens. Triazolo and Tetrazolo Pyrimidine Derivatives as HNE Inhibitors for Treating COPD. US9359362.
